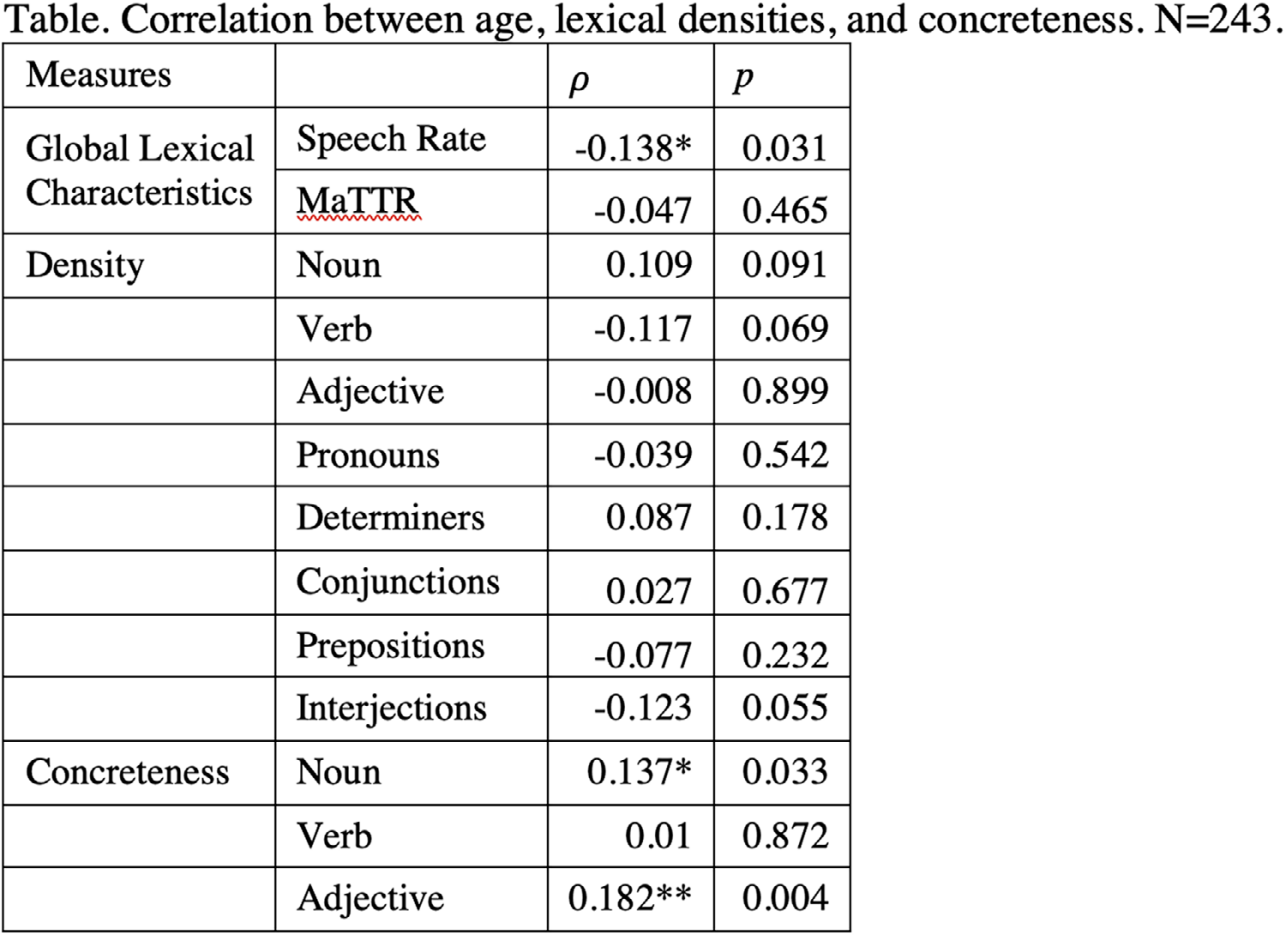# Setting a Baseline for Normal Cognitive Aging with Lexical Concreteness – A Corpus Linguistic Approach

**DOI:** 10.1002/alz.094231

**Published:** 2025-01-09

**Authors:** Kunmei Han

**Affiliations:** ^1^ National University of Singapore, Singapore Singapore

## Abstract

**Background:**

Identifying language variation in healthy aging speakers is important for understanding normal cognitive aging. Setting a baseline of normal aging languages in the first place is necessary for the evaluation of language performances of old adults. Lexical concreteness, a well‐studied psycholinguistic parameter, has been used to detect semantic memory‐related deficits. For example, people with semantic dementia were found to display reduced lexical concreteness (Breedin et al., 1994). Healthy old adults, however, are less studied in terms of the concreteness pattern in their speech. This study examines lexical concreteness of free speech samples of normal aging speakers age between 60 – 80 in Singapore.

**Method:**

We collected about 15‐min free speech from 243 English‐speaking, cognitively healthy Singaporeans (133 women, 110 men) age above 60 years old. Demographic variables (age, gender, education, and number of languages spoken) were uncorrelated with each other. Speeches were transcribed by native Singapore English speakers and tagged with part‐of‐speech (PoS) information. We calculated global lexical features including speech rate (in minutes), lexical diversity (Moving‐Average Type‐Token Ratio), and PoS distribution (count per 100 tokens). Then we calculated concreteness (Brysbaert et al., 2014) of major content words for every individual. The relation between age and lexical measures was examined with Spearman’s correlation test.

**Result:**

Results of the statistical analysis show that healthy old adults speak slower (𝜌 = ‐0.14, p = .03) with more concrete nouns (𝜌 = 0.14, p = .03) and adjectives (𝜌 = 0.18, p< .05) than younger speakers. PoS distribution and verb concreteness (all p> .05) remain stable in the aging process.

**Conclusion:**

Our speech sample revealed a general trend of elderly speech – reduced speech rates and increased lexical concreteness. The significant positive association between age and lexical concreteness in free speeches of health old adults was found for the first time, providing corroborative evidence for the link between concreteness and semantic memory. We also found a dissociation between nouns and verbs, consistent with neuropsychological literatures that nouns and verbs are encoded in different areas of the brain (Vigliocco et al., 2011). In sum, measuring lexical concreteness in natural speech data can provide circumstantial evidence for evaluating cognitive aging.